# Exploratory Laparotomy After Routine Cardiac Surgery: Results From 17,000 Patients

**DOI:** 10.1002/wjs.70341

**Published:** 2026-03-26

**Authors:** Megan Turner, James A. Brown, Pyongsoo Yoon, Michelle Yoon, Zeyu Liu, Zihan Feng, Ryan Rivosecchi, Holt Murray, Jianhui Zhu, Floyd Thoma, Derek Serna‐Gallegos, David Kaczorowski, Johannes Bonatti, Danny Chu, Irsa Hasan, Takuya Ogami, Ibrahim Sultan

**Affiliations:** ^1^ Division of Cardiac Surgery Department of Cardiothoracic Surgery University of Pittsburgh Pittsburgh Pennsylvania USA; ^2^ Heart and Vascular Institute University of Pittsburgh Medical Center Pittsburgh Pennsylvania USA; ^3^ Department of Critical Care Medicine University of Pittsburgh Pittsburgh Pennsylvania USA

**Keywords:** aortic valve replacement, coronary artery bypass grafting, exploratory laparotomy, mitral valve surgery, outcomes, perioperative care

## Abstract

**Objective:**

This study sought to elucidate the incidence and risk factors for exploratory laparotomy (“exlap”) after cardiac surgery, its associated perioperative complications, and its impact on long‐term survival.

**Methods:**

This was an observational study of consecutive STS index cardiac operations between 2010–2022, excluding circulatory arrest, transplant, and multivalvular cases. The cohort was dichotomized by the incidence of exlap in the postoperative period.

**Results:**

A total of 17,362 patients were included, of which 77 (0.5%) underwent an exlap. Postoperatively, the exlap group required more intra‐aortic balloon pumps (IABP), vasopressors, and rescue therapy (i.e., methylene blue or hydroxocobalamin) than the no‐exlap group. The exlap group had higher operative mortality, stroke, prolonged ventilation, sepsis, dialysis, mediastinal re‐exploration, and pRBC transfusions. On multivariable logistic regression, total number of postoperative pressors, use of rescue therapy, preoperative IABP, white race, chronic dialysis use, and history of cerebrovascular accident were associated with the need for postoperative exlap. On multivariable logistic regression, exlap was an independent risk factor for operative mortality (OR 3.67, 95% CI: 1.95–6.88, *p* < 0.001). Among patients surviving to discharge, Kaplan–Meier survival estimates were lower in the exlap group compared to the no‐exlap group (*p* < 0.001), with 5‐year survival being 58.9% (42.6–74.2) in the exlap group versus 84.4% (83.8–85.0) in the no‐exlap group.

**Conclusions:**

Exploratory laparotomy is rare after open‐heart surgery, and it was performed for gastrointestinal complications that occurred in the context of other severe postoperative complications. Several factors were associated with the need for exlap, including increasing age, pre‐existing renal and vascular disease, and pre‐ and post‐operative shock.

## Graphical abstract

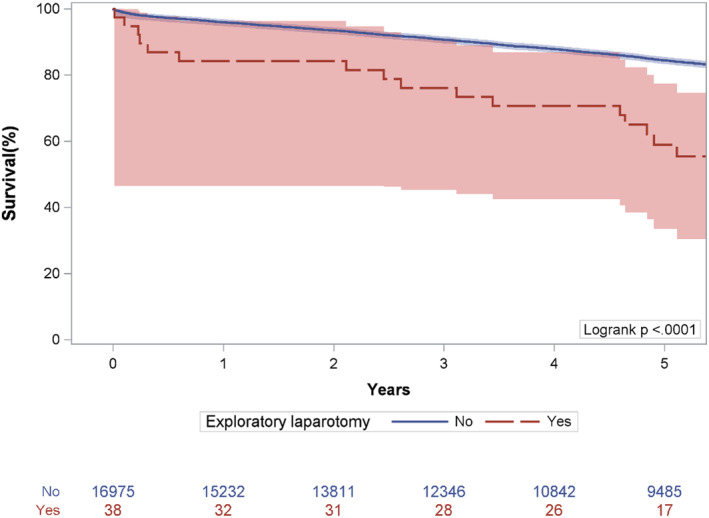

## 1 Introduction

Gastrointestinal complications (GIC) following cardiac surgery are relatively rare but have a significant impact on patient morbidity and mortality. The incidence of GIC is reported between 0.5% and 5% [1, 2, 3, 4, 5, 6, 7, 8, 9, 10, 11], depending on the type of index cardiac surgery case. Mortality after cardiac surgery complicated by GIC is reported between 10.4% and 33%, with some studies as high as 60%–70% [1, 4, 5, 6, 7, 9, 10, 11, 12, 13, 14, 15]. For those that survive, discharge to facility is much more likely [7]. Cardiac surgery patients may be at higher risk of GIC due to splanchnic hypoperfusion, resulting in poor motility, compromised mucosal integrity, ulcer formation/bleeding, and/or ischemia [2, 3, 15]. Hypoperfusion may be a result of redistribution of splanchnic circulation to vital organs in shock states, alterations to normal intestinal flow on cardiopulmonary bypass (CPB), vasodilation due to systemic inflammation from CPB, high pressor requirements due to shock states, and/or emboli secondary to aortic manipulation or postoperative arrhythmias [2, 3, 14]. Long‐term ventilator requirements, especially in the setting of high PEEP, have also been theorized to lead to gut malperfusion by perpetuating splanchnic venous congestion [2].

While many GIC may be managed conservatively or with minimally invasive procedures (i.e., scopes, drain placement) in most cases (e.g., ileus, GIB, acute cholecystitis), the complications that are most time‐sensitive and life‐threatening (i.e., mesenteric ischemia, perforated viscous) often require emergent exploratory laparotomy (“exlap”), which has been associated with very high 30‐day mortality [16]. Mortality after exlap is higher than mortality after any GIC, with estimates as high as 40%–69% [2, 4, 5, 9, 14, 17, 18, 19, 20, 21]. Invariably, some patients undergoing exlap after cardiac surgery may have negative findings, but exlap has nonetheless been associated with additional morbidity and mortality despite negative findings. However, the incidence, risk factors, and short‐term and long‐term impact of exlap after cardiac surgery remains incompletely characterized.

## 2 Patients and Methods

### 2.1 Patient Population and Study Design

This was an observational study utilizing a prospectively maintained institutional database of all consecutive cardiac operations performed at a single institution between 2010 and 2022. Definitions were consistent with the Society of Thoracic Surgeons database. All patients undergoing coronary bypass grafting (CABG), aortic valve replacement (AVR), mitral valve (MV) repair, MV replacement, CABG with AVR, CABG with MV repair, and CABG with MV replacement were included for analysis. Circulatory arrest cases, cardiac transplants, and multivalvular cases were excluded. The cohort was dichotomized by the incidence of exploratory laparotomy (“exlap”) in the postoperative period. The postoperative period was measured in days, and it spanned from the conclusion of the index cardiac operation until discharge or death.

An exlap was performed if the patient had acute‐onset hemodynamic instability (increasing vasopressor requirements), along with biochemical evidence of organ malperfusion (lactic acidosis and/or leukocytosis), in the setting of a clear change in the patient's abdominal exam (distention, pain, peritonitis, and/or GI bleeding). These combined criteria were sufficient for immediate exlap without further diagnostic imaging or procedures if the patient was too unstable for further work‐up. However, if the patient was stable enough despite their clinical deterioration, further diagnostic work‐up was preferred. Preferentially, patients underwent a computed tomography (CT) scan to ascertain a radiographic correlate of intra‐abdominal pathology (pneumoperitoneum, pneumatosis, portal venous gas, bowel wall thickening, bowel obstruction, etc.). However, patients may have had an endoscopic procedure prior to exlap if GI bleeding was the primary concern. Patients who had radiographic imaging may have had a diagnostic laparoscopy prior to exlap if the patient was stable enough to tolerate abdominal insufflation and if there was no definitive radiographic evidence of intestinal ischemia or pneumoperitoneum—for example, if there was concern for bowel obstruction or thickened bowel on imaging. Otherwise, no other diagnostic procedures were performed prior to exlap.

This study aims to determine the incidence of exlap after routine adult cardiac surgery, the risk factors for needing an exlap following cardiac surgery, its associated perioperative complications, and its impact on long‐term survival. This study was approved by the Institutional Review Board on 4/17/2019 (STUDY18120143), with written consent waived.

### 2.2 Statistical Methods and Analysis

Continuous variables were presented as mean ± standard deviation for normally distributed data, or median and interquartile range (IQR) for non‐normally distributed data. Categorical data were summarized using frequency and percentage. Student's t‐test was used to compare normally distributed continuous variables between groups, and the nonparametric Mann‐Whitney *U* test was used for non‐normally distributed continuous variables. The chi‐squared or Fisher's exact test was used to compare categorical variables between groups, as appropriate. All tests were 2‐sided with an alpha level of 0.05 considered to indicate statistical significance. Multivariable logistic regression models were built to identify variables associated with postoperative exlap and operative mortality. Unadjusted survival estimates were generated using Kaplan–Meier methods and compared using log‐rank statistics. All statistical analyses were performed using SAS/STAT Version 15.2 (SAS Institute Inc., Cary, NC, USA).

## 3 Results

### 3.1 Baseline Variables and Postoperative Outcomes

A total of 17,362 patients were included for analysis, of which 77 (0.5%) underwent a postoperative exlap. 65 of the 77 exlap patients (84.4%) had a CT scan performed prior to exlap. 3 exlap patients (3.9%) had an endoscopic procedure for lower GI bleeding that was ultimately uncontrollable or unlocalizable endoscopically, resulting in exlap, total abdominal colectomy, and end ileostomy creation. 14 patients (18.2%) underwent a diagnostic laparoscopy prior to exlap. 10 patients (13.0%) had a negative exlap. Positive findings included any intra‐abdominal pathology (especially bowel ischemia, necrosis, perforation, or obstruction) requiring operative intervention (bowel resection/repair and/or vascular intervention), as well as any intra‐abdominal pathology deemed non‐survivable (whether any operative intervention was performed or not). A negative exlap was declared if there was no intra‐abdominal pathology discovered after running the entire bowel and inspecting the remaining intra‐abdominal organs.

Table 1 compares baseline variables across each group. Table 2 shows postoperative outcomes for all patients who underwent exlap, while Supporting Information S1: Table S1 compares postoperative outcomes across each group.

**TABLE 1:** Demographic and baseline characteristics.

Variable	No exploratory laparotomy (*n* = 17,285)	Exploratory laparotomy (*n* = 77)	*p*‐value
Age (years)	66.5 ± 10.8	70.1 ± 10.9	0.004
Female	4907 (28.4)	20 (26.0)	0.64
White race	11670 (67.5)	60 (77.9)	0.05
Body mass index (kg/m^2^)	30.1 ± 6.2	29.5 ± 7.41	0.37
Diabetes	7339 (42.5)	31 (40.3)	0.70
Chronic dialysis use	405 (2.3)	9 (11.7)	< 0.001
Severe chronic lung disease	464 (2.7)	4 (5.2)	0.18
Peripheral vascular disease	2758 (16.0)	16 (20.8)	0.25
Prior cerebrovascular accident	1383 (8.0)	14 (18.2)	0.001
Hematocrit	39.0 ± 5.4	36.7 ± 6.5	< 0.001
Creatinine	1.17 ± 0.96	1.72 ± 1.56	< 0.001
Atrial fibrillation	2218 (12.8)	13 (16.9)	0.29
Congestive heart failure	3768 (21.8)	24 (31.2)	0.05
Preoperative inotrope	309 (1.8)	5 (6.5)	0.002
Preoperative IABP	766 (4.4)	12 (15.6)	< 0.001
Ejection fraction	52.2 ± 12.1	49.8 ± 14.2	0.08
Redo cardiac surgery	1066 (6.2)	7 (9.1)	0.29
STS‐PROM	3.0 ± 4.0	8.0 ± 10.0	< 0.001
Surgical status			< 0.001
Elective	8566 (49.6)	28 (36.4)	
Urgent	8222 (47.6)	41 (53.3)	
Emergent/salvage	497 (2.9)	8 (10.4)	
Surgical procedure			< 0.001
CABG	11279 (65.3)	37 (48.1)	
MV repair	811 (4.7)	1 (1.3)	
MV replacement	430 (2.5)	7 (9.1)	
AVR	2461 (14.2)	8 (10.4)	
CABG + AVR	1719 (10.0)	16 (20.8)	
CABG + MVrepair	423 (2.5)	3 (3.9)	
CABG + MVreplacement	162 (0.9)	5 (6.5)	

**TABLE 2:** Outcomes among patients who had an exploratory laparotomy.

Variable	Exploratory laparotomy (*n* = 77)
Operative mortality (STS definition)	19 (24.7)
Stroke	6 (7.8)
Total number of postoperative pressors^a^	3.2 ± 1.4
Postoperative rescue therapy^b^	9 (11.7)
Intraoperative IABP	7 (9.1)
Postoperative IABP	2 (2.6)
Prolonged ventilation (> 24 h)	60 (77.9)
Sepsis	20 (26.0)
New dialysis requirement	28 (36.4)
Re‐exploration for bleeding	6 (7.8)
pRBC transfusion	65 (84.4)
Length of stay	24.0 [14.0–35.0]

^a^ epinepherine, norepinephrine, vasopressin, dopamine, phenylephrine.

^b^ methylene blue or hydroxocobalamin.

Time to exlap in the overall cohort was 9.2 ± 9.1 days. To determine if the time to exlap was associated with mortality, Student's *t*‐test was used to compare time to exlap among patients who died versus patients who survived to discharge. In the exlap group alone, patients who survived to discharge had a time to exlap of 9.3 ± 8.6 days, while patients with in‐hospital mortality had a time to exlap of 8.8 ± 10.9 days (*t*‐test, *p* = 0.847).

### 3.2 Regression Models for Exlap and Operative Mortality

Table 3 shows a multivariable logistic regression model of risk factors associated with exlap. Total number of postoperative pressors, use of rescue therapy, preoperative IABP, white race, chronic dialysis use, and history of cerebrovascular accident were associated with the need for postoperative exlap.

**TABLE 3:** Multivariable logistic regression for postoperative exploratory laparotomy.

Variable	OR	95% CI	*p*‐value
Age	1.02	0.99–1.04	0.11
Total number of pressors	3.31	2.73–4.01	< 0.001
White race	2.19	1.24–3.87	0.007
Chronic dialysis use	3.70	1.72–8.00	< 0.001
Prior cerebrovascular accident	2.23	1.21–4.12	0.01
Rescue therapy	3.43	1.53–7.66	0.003
Preoperative IABP	2.01	1.03–3.93	0.04
Diabetes	0.70	0.43–1.14	0.15

Table 4 shows a logistic regression model for operative mortality. On multivariable logistic regression, exlap was an independent risk factor for operative mortality (OR 3.67, 95% CI: 1.95–6.88, *p* < 0.001).

**TABLE 4:** Multivariable logistic regression for operative mortality.

Variable	OR	95% CI		*p*‐value
Exploratory laparotomy	3.67	1.95	6.88	< 0.001
Age	1.04	1.02	1.05	< 0.001
White race	2.43	1.73	3.43	< 0.001
Ejection fraction	0.99	0.98	1.00	0.04
Number of total pressors	2.28	2.05	2.53	< 0.001
Diabetes	1.30	1.01	1.69	0.05
Chronic dialysis use	2.39	1.44	3.95	< 0.001
Peripheral vascular disease	1.54	1.17	2.04	0.003
Prior cerebrovascular accident	1.38	0.96	2.00	0.09
Congestive heart failure	1.74	1.32	2.29	< 0.001
Redo cardiac surgery	1.85	1.29	2.65	< 0.001
Emergent (ref: elective)	3.04	2.05	4.51	< 0.001
CABG + MV repair (ref: CABG)	1.61	1.00	2.61	0.05
Aortic valve replacement	0.69	0.43	1.09	0.11
Hematocrit	0.98	0.95	1.00	0.03

### 3.3 Long‐Term Survival

**FIGURE 1:**
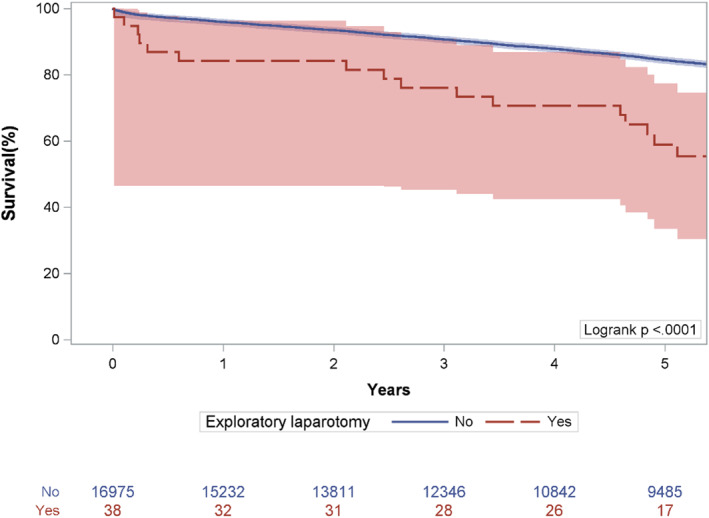
Kaplan–Meier survival estimates among patients surviving to discharge, compared across patients who did and did not undergo a postoperative exploratory laparotomy.

Median [IQR] follow‐up was 5.6 [2.5–8.9] years. Figure 1 (and *Central Picture*) shows Kaplan–Meier survival estimates for patients who survived to discharge, compared across each group. Among patients surviving to discharge, Kaplan–Meier survival estimates were lower in the exlap group compared to the no exlap group (*p* < 0.001), with 5‐year survival being 58.9% (42.6–74.2) in the exlap group versus 84.4% (83.8–85.0) in the non‐exlap group.

## 4 Comment

In this study of exlap after cardiac surgery, a few notable findings were evident. Exlap was rare in this cohort of STS index operations, occurring in only 0.5% of patients. However, the exlap group had significantly more postoperative complications than the non‐exlap group, including total number of pressors, rescue therapy, IABP, stroke, prolonged ventilation, new dialysis requirement, re‐exploration of bleeding, pRBC transfusions, and sepsis. Most notably, operative mortality in the exlap group was significantly higher than the non‐exlap group (24.7% vs. 1.5%), with exlap conferring 3.7 increased odds of operative mortality on multivariable logistic regression. Several factors were associated with the need for exlap, including increasing age, pre‐existing renal and vascular disease, and pre‐ and post‐operative shock. Finally, exlap was associated with reduced long‐term survival, even among patients surviving to discharge, suggesting that the adverse impact of exlap extends into the long run. By implication, exlap is a rare, but serious, complication of open‐heart surgery. Even though some risk factors for exlap may be non‐modifiable, avoidance of perioperative shock may mitigate the risk of exlap and its devastating consequences.

The risk factors for exlap identified in the present analysis are consistent with previous studies. Advanced age, pre‐existing renal failure, chronic lung disease, pre‐existing heart failure, peripheral vascular disease, diabetes, and hypertension have routinely been associated with GIC after cardiac surgery. Among operative characteristics, redo operations, emergent/salvage status, and valve surgery (vs. CABG) have been associated with GIC, while prolonged CPB time, IABP requirement, postoperative renal failure, prolonged mechanical ventilation, postoperative arrhythmia, stroke, reoperation for bleeding, and blood transfusions have been shown to be perioperative risk factors [1, 2, 3, 7, 8, 10, 11, 12, 14, 21]. Additionally, data evaluating risk factors specifically for mesenteric ischemia have cited similar variables [2, 4, 14, 17, 18, 19, 20, 22, 23], with shock states being particularly associated with mesenteric ischemia, as reflected by high doses of pressors and inotropes [2, 14, 17, 20, 22, 23]. In this study, chronic dialysis use, prior CVA, preoperative IABP, total number of postoperative pressors, and need for rescue therapy were significantly associated with exlap. Chronic dialysis use, total number of pressors, and need for rescue therapy had the highest increased odds of exlap, with odds ratios > 3. In the setting of perioperative shock, splanchnic malperfusion leading to mesenteric ischemia may be the most significant predictor of the need for an exlap.

In this study, the operative mortality of patients undergoing exlap is consistent with prior studies focusing on exlap for mesenteric ischemia after cardiac surgery [2, 4, 5, 9, 14, 17, 18, 19, 20]. Exlap was the strongest independent predictor of operative mortality in this study's patient population with an odds ratio of 3.7. Abboud et al. reported that mesenteric ischemia after cardiac surgery was associated with a mortality rate of 42.8% for patients with positive exlaps, and 50% for those with negative exlap, although this was limited by small sample size (*n* = 13, 0.8% of cohort) [17]. Similarly, this study also found that the adverse impact of exlap extends into the long run. Among patients surviving to discharge, the exlap group had significantly reduced overall survival compared to the non‐exlap group. This survival difference among patients surviving to discharge may be due to more comorbidities in the exlap group, rather than exlap itself having an independent impact on survival. However, exlap was also associated with a number of other postoperative complications, including stroke, prolonged ventilation, new dialysis requirement, re‐exploration of bleeding, pRBC transfusions, and sepsis—all of which have been independently associated with reduced survival. Thus, even though the exlap group had more baseline comorbidities than the non‐exlap group, exlap is associated with devastating consequences that are likely to reduce survival in the long‐term. Similarly, prior data evaluating long‐term survival of patients experiencing noncardiac postoperative complications after cardiac surgery, including GIC, respiratory failure, sepsis, renal failure requiring dialysis, mediastinitis, and stroke, reported < 50% survival at 1‐year among patients with ≥ 3 complications [24].

Exlap post cardiac surgery is undoubtedly a devastating complication and incurs its own risks and complications to an already critically ill patient. However, early exlap in the appropriate clinical scenario does confer a survival benefit. A study by Ariyaratnam et al. (2015) looked at patients who developed bowel ischemia following cardiac surgery (*n* = 85)—60% underwent laparotomy, with an in‐hospital mortality of 39.2% versus 91.2% for those who did not undergo laparotomy [18]. Similarly, in a study by Filsoufi et al. (2007) looking at mesenteric ischemia in cardiac surgery patients (*n* = 30), 53% underwent exlap, with a mortality rate of 25% for those who underwent intervention and 71% for those without [6]. Likely those who did not undergo surgical intervention had either a delay in diagnosis or were considered too ill to pursue treatment [6]. Still, Elgharably et al. (2021) reported similar mortality rates regardless of whether exlap was pursued in patients diagnosed with mesenteric ischemia (*n* = 104)—45% versus 46%—with no reported difference in time to diagnosis or intervention [5]. Many of these studies are limited given the low incidence and thus low sample size, however, it does raise the question of whether there are specific criteria that could be used to guide aggressiveness of intervention when exlap is considered, when exlap itself is an independent predictor of mortality.

### 4.1 Limitations

There are several important limitations to this study. As a retrospective analysis of existing observational data, each group may have been confounded by differences in the distribution of baseline comorbidities. Moreover, small sample size may have limited the power of this study to detect differences across each group. Specifically, to further elucidate the impact of exlap per se, it would be necessary to compare outcomes across patients with a negative exlap versus patients with a positive exlap to determine if patients that ultimately had intra‐abdominal pathology fared worse than their counterparts with negative findings during exlap. However, the number of patients with a negative exlap was too small (*n* = 10) for statistical analysis. Similarly, due to the retrospective nature of this study, it is unknown how many patients in the non exlap group may have suffered a significant GI complication that would have benefited from a timely exlap, which would have provided a meaningful comparator group of analysis. Finally, some relevant variables were not available for analysis, including the presence and severity of visceral arteriosclerosis.

## 5 Conclusion

Exploratory laparotomy is rare after open‐heart surgery and was associated with increased morbidity and mortality. Occurring in 0.5% of routine cardiac surgery patients, exlap was associated with more postoperative complications, including operative mortality, perioperative shock, stroke, prolonged ventilation, dialysis, re‐exploration of bleeding, pRBC transfusions, and sepsis. Several factors were associated with the need for exlap, including increasing age, pre‐existing renal and vascular disease, and pre‐ and post‐operative shock. Finally, exlap was associated with reduced long‐term survival, even among patients surviving to discharge, suggesting that the adverse impact of exlap extends into the long run. Even though some risk factors for exlap may be non‐modifiable, avoidance of perioperative shock may mitigate the risk of exlap and its devastating consequences.

## Author Contributions

**Megan Turner:** conceptualization, investigation, writing – original draft, methodology, writing – review and editing, formal analysis, data curation. **James A. Brown:** conceptualization, investigation, writing – original draft, methodology, writing – review and editing, formal analysis, supervision. **Pyongsoo Yoon:** conceptualization, investigation, writing – original draft, methodology, writing – review and editing, formal analysis, supervision. **Michelle Yoon:** conceptualization, investigation, writing – original draft, methodology, writing – review and editing, formal analysis, data curation. **Zeyu Liu:** conceptualization, investigation, writing – original draft, methodology, writing – review and editing, formal analysis, data curation. **Zihan Feng:** conceptualization, investigation, writing – original draft, methodology, writing – review and editing, formal analysis, data curation. **Ryan Rivosecchi:** conceptualization, investigation, writing – original draft, methodology, writing – review and editing, formal analysis, supervision. **Holt Murray:** conceptualization, investigation, writing – original draft, methodology, writing – review and editing, formal analysis, supervision. **Jianhui Zhu:** conceptualization, investigation, writing – original draft, methodology, writing – review and editing, formal analysis, data curation. **Floyd Thoma:** conceptualization, investigation, writing – original draft, methodology, writing – review and editing, formal analysis, data curation. **Derek Serna‐Gallegos:** supervision, formal analysis, methodology, writing – review and editing, conceptualization, investigation, writing – original draft. **David Kaczorowski:** conceptualization, investigation, writing – original draft, methodology, writing – review and editing, formal analysis, supervision. **Johannes Bonatti:** supervision, formal analysis, methodology, writing – review and editing, writing – original draft, investigation, conceptualization. **Danny Chu:** supervision, formal analysis, methodology, writing – review and editing, writing – original draft, investigation, conceptualization. **Irsa Hasan:** supervision, formal analysis, methodology, writing – review and editing, writing – original draft, investigation, conceptualization. **Takuya Ogami:** conceptualization, investigation, writing – original draft, methodology, writing – review and editing, formal analysis, supervision. **Ibrahim Sultan:** conceptualization, writing – original draft, investigation, methodology, writing – review and editing, formal analysis, supervision.

## Funding

The authors have nothing to report.

## Conflicts of Interest

The authors declare no conflicts of interest.

## Supporting information


Table S1

## Data Availability

The data that support the findings of this study are available on request from the corresponding author. The data are not publicly available due to privacy or ethical restrictions.
