# Supplementary material for: Exploratory Laparotomy After Routine Cardiac Surgery: Results From 17,000 Patients
Source: World J Surg. 2026 Mar 26;50(5):1221–7. doi: 10.1002/wjs.70341 (PMC13206261; doi:10.1002/wjs.70341)
Supplement: Supplementary file 1 — Table S1 [file WJS-50-1221-s001.docx]

**Supplemental Table 1. Outcomes compared across each group**

| **Variable** | **No exploratory laparotomy (n=17,285)** | **Exploratory**  **laparotomy (n=77)** | **p-value** |
| --- | --- | --- | --- |
| Operative mortality (STS definition) | 259 (1.5) | 19 (24.7) | <0.001 |
| Stroke | 274 (1.6) | 6 (7.8) | <0.001 |
| Total number of postoperative pressors* | 1.2 ± 1.1 | 3.2 ± 1.4 | <0.001 |
| Postoperative rescue therapy° | 132 (0.8) | 9 (11.7) | <0.001 |
| Intraoperative IABP | 282 (1.6) | 7 (9.1) | <0.001 |
| Postoperative IABP | 72 (0.4) | 2 (2.6) | 0.04 |
| Prolonged ventilation (>24 hours) | 1338 (7.7) | 60 (77.9) | <0.001 |
| Sepsis | 112 (0.7) | 20 (26.0) | <0.001 |
| New dialysis requirement | 303 (1.8) | 28 (36.4) | <0.001 |
| Re-exploration for bleeding | 436 (2.5) | 6 (7.8) | 0.003 |
| pRBC transfusion | 5343 (30.9) | 65 (84.4) | <0.001 |
| Length of stay | 8.0 [6.0-11.0] | 24.0 [14.0-35.0] | <0.001 |

* epinepherine, norepinephrine, vasopressin, dopamine, phenylephrine

° methylene blue or hydroxocobalamin
